# Characterization of *bhatooru,* a traditional fermented food of Himachal Pradesh: microbiological and biochemical aspects

**DOI:** 10.1007/s13205-012-0092-2

**Published:** 2012-09-18

**Authors:** Savitri, T. C. Bhalla

**Affiliations:** Department of Biotechnology, Himachal Pradesh University, Summerhill, Shimla, 171005 Himachal Pradesh India

**Keywords:** *Bhatooru*, Traditional fermented foods, Himachal Pradesh, Fermentation

## Abstract

A number of traditional fermented products are prepared and consumed in Himachal and the types of traditional fermented products of Himachal are unique and different from other areas. *Bhatooru* is an indigenous leavened bread or *roti* and constitutes the staple diet of rural population of Himachal. The microbiological analysis of the inoculums (*malera*) revealed that it composed of a consortium of microorganisms. Population of *Lactobacillus, Leuconostoc* and *Saccharomyces cerevisiae* increased from 4.77 to 8.0 log cfu/g of dry matter in 10 h of fermentation. The amount of total proteins increased from 13.6 to 18.4 % (w/w). The total sugars during fermentation decreased from 74.1 to 50.1 % (w/w) on dry weight basis. However, the reducing sugar level of the fermenting samples increased significantly from 7.8 to 16.5 mg/g dry matter in the first 4 h and thereafter, it gradually decreased to 10.0 mg/g dry matter. Similarly starch content decreased from 70.2 to 48.3 % (w/w) on dry weight basis by 10 h of fermentation. In fermented samples protease activity increased from 0.48 U/g dry matter to 11.5 U/g in 6 h and then decreased to 3.21 U/g on dry weight basis at 10 h. Amylase activity initially increased from 65.0 to 79.4 U to 6 h and then declined to 69.9 U/g of dry matter. Fermentation in *bhatooru* significantly enhanced the B vitamin levels especially thiamine, riboflavin and nicotinic acid and essential amino acids viz methionine, phenylalanine, threonine, lysine and leucine.

## Introduction

Fermentation is one of the oldest methods of food preservation and is widely practiced at household level by rural folk to produce variety of traditional fermented foods and beverages (Cooke et al. 1987; Sasson 1988). Fermented foods generally preserve pleasant flavor, aroma, texture, enhanced nutritive values and good keeping quality under ambient conditions (Law et al. 2011). Several indigenous fermented foods and beverages produced at the household level in Swaziland were reviewed by Masarirambi et al. (2009). India being a large country displays climatic, ethnic and religious diversities vis-à-vis variation in food production and consumption. A lot of diversity prevails in the food habits of the people living in different parts of the country especially in the hilly regions where people have evolved indigenous method of preparing fermented foods and beverages based on easily available local raw materials. The skills of food preservation existed in the native people and the know-how of these fermentation was propagated orally (Prajapati 2003). Diversity of fermented foods in Asia is directly related to food culture of each and every community, and also the availability of raw materials (Tamang 2011).

In Himachal Pradesh, people have developed traditional food processing technologies for preparing fermented foods from locally available substrates largely governed by the ethnic preference, agroclimatic conditions, socio-cultural ethos and religion. A number of traditional fermented products are prepared and consumed in Himachal and the types of traditional fermented products of Himachal are unique and different from other areas (Thakur et al. 2003). *Bhatooru, chilra, seera, siddu, gulgule, marchu, sepubari* and pickles made from various locally available fruits and vegetables and different beverages like *kinnauri, chhang, sura, behmi*, etc. are some indigenous fermented products of Himachal Pradesh (Savitri 2007). Fermented foods have been a part of the staple diet in the rural areas of Himachal (specially the districts of Lahaul and Spiti, Kinnaur, Chamba and Kullu).

*Bhatooru* is indigenous leavened bread that contributes the staple diet of rural population of Himachal. *Bhatooru* is served with vegetables, *dal* or curry for routine meals and deep-fried on festive occasions. *Malera* is a traditional inoculum used for preparation of *bhatooru.*

## Materials and methods

The samples of the inoculum (*malera*) used for *bhatooru* fermentation were collected aseptically from different areas of the state, stored in refrigerator and used for further studies. Wheat flour for preparation of *bhatooru* was purchased from the local market of Shimla, Himachal Pradesh (‘*Shakti Bhog* brand’).

### Preparation of *bhatooru*

*Bhatooru* was prepared by mixing 450 g of wheat flour with 300 ml of water. 50 g of *malera* (traditional inoculum) was added to the mixture and knead to form consistent dough. The dough was kept at 25 °C in incubator for 10 h for fermentation. After every 2 h, samples were taken and stored at 4 °C for further analysis. For microbiological analysis, the samples were processed immediately. A part of the dough was baked to prepare *bhatooru.*

### Microbial profile during the fermentation

One gram of *malera* and one gram of dough sample withdrawn at an interval of 2 h were plated on nutrient agar, Czapek Malt agar and *Lactobacillus* selection agar plates for isolation of bacteria, yeasts and lactic acid bacteria by incubating at 30 °C for bacteria and 25 °C for yeasts. The number of colonies of bacteria and yeasts that appeared on plates after 24–48 h of incubation were counted and expressed as cfu g^−1^ of the sample. The microorganisms isolated were identified at and submitted to Microbial Type Culture Collection and Gene Bank (MTCC), Chandigarh.

### Biochemical analysis

Samples during *bhatooru* dough fermentation were analyzed for various biochemical parameters viz., moisture by Winton and Winton (2001), total acidity by Amerine et al. (1980) and pH. Total proteins were estimated by using the methods of Lowry et al. (1951) and total carbohydrates were estimated by phenol sulphuric acid method (Dubois et al. 1956). Reducing sugars were estimated by DNSA method given by Miller (1959). Starch was estimated according to Hedge and Hofreiter (1962). The activity of protease and amylase was assayed by the method given by Manachini et al. (1988) and Bernfield (1955), respectively. SDS polyacrylamide gel electrophoresis of the proteins of fermented dough has also been performed to analyze the protein profile (gliadin and glutenin) during *bhatooru* fermentation.

### Vitamin and amino acid analysis

Analysis of vitamins in fermented dough, *malera* and flour has been done according to Šnajdrová et al. (2004). For assay of water soluble B vitamins, the samples of fermented food were filtered through 0.45 μm pore size filters. The mobile phase composed of acetonitrile:HPLC water (75:25) and 0.1 % orthophosphoric acid. Samples (5 μl) of the solution of water-soluble vitamins were injected into the HPLC column. Identification of compounds was made by comparing their retention times and UV spectra with those of standards. The vitamin concentrations in the samples were calculated from the integrated areas of the samples and their corresponding standards.

For amino acid analysis, sample was first hydrolysed (Schilling et al. 1996) and then derivatization (Hûsek 1991) was done. Prior to analysis, the samples were dried at room temperature for 40 h. One gram sample was taken and transferred to glass sample tubes. These samples were placed inside a glass hydrolysis chamber. To each tube, 2 μl of norleucine was added as internal standard solution (1,500 ppm in 0.1 M HCl). A 200 μl of 6 M HCl was introduced to the bottom of the chamber and kept at 105 °C in an oven for 24 h to fully hydrolyze the samples. After complete hydrolysis, the remaining traces of acid were removed by washing with 15 μl of water and then dried at 50 °C. The hydrolysate was dissolved in 120 μl of 25 mM HCl.

An aliquot of the hydrolysate was taken in a vial which usually contained less than 100 μg of amino acids. To this 100 μl of water:ethanol:pyridine (60:32:8) was added and mixed properly. Then 5 μl of ECF was added to this mixture and the tube was shaken gently for about 5 s till foaming due to gas evolution occurred. 100 μl of chloroform (containing 1 % ECF) was added and the vials were gently tapped to facilitate separation of the two layers. 2 μl of chloroform layer (lower layer) was injected in GC. Gas chromatographic analysis was carried out on a Netel Chromatograph GC (MICHRO-9100) equipped with Chromosorb WHP 15 % SE-30 column coupled with flow Ionization Detector. The GC was operated at the oven temperature 170–295 °C, injector temperature 170–280 °C, ramp rate 5 °C and carrier flow (nitrogen) 5 ml/min.

## Results and discussion

### Microbiological analysis of *malera* (traditional inoculum) and dough in bhatooru fermentation

The microbiological analysis of *malera* revealed that it was a consortium of microorganisms which mainly consisted of lactic acid bacteria and yeast. *Lactobacillus plantarum* (MTCC 8296)*, Leuconostoc* sp. and *Saccharomyces cerevisiae* (MTCC 7840) were isolated from different samples of *malera.* The microflora of *malera* depends on flour, water used for dough preparation, utensils used, prevailing hygienic conditions as well as various parameters of the fermentation. The most relevant bacteria isolated from sourdough belonged to the genus *Lactobacillus* (Stolz 2003). Various yeast strains have also been isolated from spontaneous sourdough fermentations such as *Saccharomyces cerevisiae* and *Pichia satoi* (Beech and Davenport 1971). There have been several reports (Okada et al. 1992; Oura et al. 1982; Spicher 1984; Spicher and Schroder 1978, 1980) of lactobacilli occurring among the dominant microbial population in sourdough where they contribute to dough fermentation. *Lactobacillus* species are widely distributed in various fermented foods, dairy products and plant and animal materials (Cai et al. 1999).

A large number of bacteria and yeast were isolated from fermented dough samples of *bhatooru* fermentation at different intervals of time (Table 1). The microflora of the fermented dough was mainly dominated by yeast (*Saccharomyces cerevisisae*), lactic acid bacteria (*Lactobacillus plantarum*) and *Bacillus* sp. The gas producing *Leuconostoc* sp. also appeared at 4 h of fermentation causing leavening of dough. The source of these organisms might be the ingredients, vessels, and the surroundings followed by rapid multiplication during fermentation. With the progress in fermentation, total microbial count increased from 6 × 10^4^ to 1 × 10^8^ cfu/g decreasing the pH from 5.94 to 4.18. The decrease in the pH prevents the growth of undesirable microorganisms but the desirable microorganisms like yeast, *Leuconostoc* and *Lactobacilli* can very well propagate at this pH. *Saccharomyces cerevisisae* has been reported from various fermented foods and beverages such as *bhalle*, beer*, burukutu,* bourbon whiskey, coffee beans, cider, *merissa, fufu, tape, ogi, puto, dosa, idli, papdam, kecap, lao chao*, *warri,* scotch whiskey, etc. (Padmaja and George 1999; Batra and Millner 1974, 1976; Soni and Sandhu 1990). Some species of *Bacillus* and other bacteria such as *Kocuria rhizophila, Pseudomonas synxantha* and *Microbacteriun saperdae* were also found during the initial stages of *bhatooru* fermentation and these organisms gradually disappeared with the progress of fermentation. This may be due to the production of acids and gas from various carbohydrates by lactic acid bacteria thus making the environment unfit for many of the bacterial population initially present.

**Table 1:** Changes in microflora, pH and volume in dough during *bhatooru* fermentation

Incubation time (h)	Volume (ml)	Total count (log cfu/g)	Predominant microorganism
0	500	4.77	*Saccharomyces cerevisiae, Bacillus* sp., *Lactobacillus plantarum, Kocuria rhizophila*
2	510	6.69	*S. cerevisiae, Bacillus* sp., *Microbacterium saperdae, L. plantarum, Kocuria rhizophila, Pseudomonas synxantha*
4	539	6.69	*S. cerevisiae, Bacillus* sp., *L. plantarum, Leuconostoc* sp.
6	560	7.0	*S. cerevisiae, Bacillus* sp., *L. plantarum, Leuconostoc* sp.
8	583	7.56	*S. cerevisiae, L. plantarum, Leuconostoc* sp.
10	597	8.0	*S. cerevisiae, L. plantarum*

*Bacillus cereus*, *Flavobacterium* sp. and *Cellolomonas* sp. were reported during initial stages of fermentation as minor microbial flora

Dough used for *bhatooru* preparation has also been prepared without the addition of *malera* (control) to compare the fermentation process with *malera* added preparation. These studies showed the involvement of only bacteria like *Bacillus* sp., *Kocuria rhizophila* and *Enterobacter* sp. in control and the total count of bacterial population increases from 4 × 10^2^ cfu/g at 0 h to 5 × 10^6^ cfu/g of dry matter at 10th h. Microorganisms (*Leuconostoc* sp., *Lactobacillus* sp. and *S. cerevisiae*) which actively produce gases and acids were absent in the control dough (Table 1). Moreover, such preparation lacked the typical aroma otherwise contributed by the yeast and lactic acid bacteria in the fermented product.

### Chemical and biochemical analysis of fermented dough

The traditional inoculum-*malera* used for *bhatooru* fermentation was collected from Kullu and analyzed for various biochemical parameters. The biochemical analysis of *malera* revealed that it is an acidic dough having a pH of 3.7, titratable acidity of 0.62 and 46 % moisture. It has 20 % (w/w) protein, 571.0 mg/g of dry matter carbohydrate, 496.0 mg/g dry matter starch and 23.2 mg/g dry matter reducing sugars. The activities of amylase and protease were 25.7 and 1.67 U/g respectively.

With the decrease in pH from 5.94 to 4.18, total acidity in fermented sample increased from 0.028 to 0.14 %. This may be due to the production of acetic acid and lactic acid during fermentation by lactic acid bacteria and yeast. The increase in acid content is in proportion with the increase in lactic acid bacteria count. The increase in the total acidity in fermented sample helps in enhancing the shelf life of fermented foods as well as it imparts typical aroma and taste to the product (Hammes and Gänzle 1998). However, the change in both pH (from 6.7 to 6.48) and acidity (from 0.018 to 0.045 %) is very less in the case of control. The fall in pH and increase in titratable acidity in dough during fermentation are considered important for prevention of malfermentation and spoilage of bread. At low pH of sourdough, the growth and activity of spoilage organisms such as *Bacillus subtilis* or *Clostridia* which cause ropiness, are suppressed (Hammes and Gänzle 1998).

The change in total protein content during *bhatooru* fermentation has been studied and it was found that there was an increase in total proteins from 13.6 to 18.4 % (w/w) on dry weight basis from 0 to 10th h of fermentation. However, there was no significant change in total protein content in case of control. Increase in protein content in fermented food on dry weight basis might be due to utilization of carbohydrates. The amount of total sugars during fermentation decreased from 74.1–50.1 % (w/w) on dry weight basis in fermented dough and 73.9–66.1 % (w/w) on dry weight basis in control dough (Table 2). The decrease in total sugars might be due to the metabolism of sugars by bacteria and yeast. The sugars are rapidly metabolized to acids, ethanol, biomass, carbon dioxide and other metabolites required for the growth of microorganisms with concomitant decrease in total sugars during fermentation (Mensah 1997).

**Table 2:** Biochemical parameters in fermented dough during *bhatooru* fermentation and control

Incubation time (h)	Protein (mg/g dry matter)		Total sugars (%)		Reducing sugars (mg/g dry matter)		Starch (%)		Amylase (μg/g/min)		Protease (μg/g/min)	
	F	C	F	C	F	C	F	C	F	C	F	C
0	13.6 ± 0.78	13.4 ± 1.40	74.1 ± 2.17	73.9 ± 1.51	7.8 ± 1.21	4.0 ± 0.96	70.2 ± 2.14	67.2 ± 0.862	65 ± 1.67	61.6 ± 1.27	0.48 ± 0.03	0.32 ± 0.04
2	14.2 ± 1.19	13.5 ± 0.93	72.5 ± 1.65	72.2 ± 1.01	16.5 ± 0.66	12.6 ± 0.8	70.9 ± 1.47	68.4 ± 1.069	68.9 ± 1.92	62.7 ± 0.92	1.35 ± 0.07	0.41 ± 0.05
4	15.8 ± 1.68	13.3 ± 0.94	70.1 ± 0.92	70.1 ± 1.87	17.2 ± 1.46	15.7 ± 1.86	69.3 ± 0.92	68.7 ± 1.210	71.8 ± 0.9	46.2 ± 1.39	8.29 ± 0.11	0.45 ± 0.03
6	16.4 ± 1.14	14.1 ± 1.66	57.2 ± 1.47	69.7 ± 1.06	15.6 ± 0.94	17.5 ± 1.07	68.6 ± 0.85	68.6 ± 1.136	77.9 ± 1.95	52.6 ± 0.92	11.59 ± 0.89	0.45 ± 0.02
8	18.3 ± 1.37	14.3 ± 1.83	54.2 ± 1.56	67.5 ± 0.87	14.4 ± 1.03	21.1 ± 0.96	54.9 ± 1.11	69.0 ± 0.986	74.2 ± 0.75	33.4 ± 0.79	6.27 ± 0.62	0.49 ± 0.03
10	18.4 ± 1.18	14.0 ± 0.56	50.1 ± 2.31	66.1 ± 0.89	10.0 ± 0.30	15.7 ± 0.36	48.3 ± 1.88	67.0 ± 1.572	69.9 ± 0.67	33.1 ± 0.65	3.21 ± 0.22	0.50 ± 0.02

Values are mean ± SD of three observations

*F* Fermented dough, *C* Control

As given in Table 2 the level of reducing sugar in dough increased significantly in the first 4 h of fermentation and thereafter it gradually decreased to 10.0 mg/g dry matter by 10 h. In control, the level of reducing sugars increased gradually from 4.0 to 21.1 mg/g dry matter up to 8 h and thereafter it declined to 15.7 mg/g dry matter at 10th h. Initial increase in reducing sugar can be implicated with the activity of inherent amylases in the flour which might have got activated with the addition of water during the preparation of dough. The rate of conversion of starch to reducing sugars mediated by amylases was initially higher than the rate of consumption of reducing sugars by the fermentative organisms and later the rate of sugar consumption got enhanced due to augmentation in the number of fermentative organisms. It has been reported that as the pH of the ferment decreases, the saccharification of starch by amylases and amyloglucosidases is also reduced and this results in gradual decrease of reducing sugars concentration towards the later stages of fermentation (Syu and Chen 1997; Narendranathan et al. 1997).

The change in starch content in dough during 10 h of *bhatooru* fermentation is given in Table 2. The starch content in dough was 70.2 % (w/w) on dry weight basis at 0 h. After 2 h of fermentation it started decreasing and finally (at 10 h fermentation) its level went down to 48.3 % (w/w) on dry weight basis. The starch content in control almost remained constant. Starch is saccharified to monosaccharides and disaccharides by inherent amylases of flour, which are utilized by lactic acid bacteria and yeast present in the sourdough for their growth and production of acids (Röcken et al. 1992).

### Protease and amylase activity

The protease activity was more in fermented dough sample as compared to that of control. In control, the activity of enzyme ranged from 0.32 to 0.50 U/g dry weight of sample while in fermented dough samples, the protease activity increased from 0.48 U/g dry matter to 11.5 U/g in 6 h and then decreased to 3.21 U/g on dry weight basis in 10 h of fermentation (Table 2). The proteolytic activity during the fermentation of sourdough leads to the enhancement of free amino acid content that are well known precursors of flavour formation in bread (Thiele et al. 2002). The proteolysis is caused by flour enzymes, microbial enzymes of flour and by sourdough bacteria. The proteolytic activity has been mainly attributed to the endogenous enzymes of the flour (Kratochvil and Holas 1988; Spicher and Nierle 1988). The lactic acid bacteria of sourdough have also been reported to produce protease during the fermentation of sourdough (Spicher and Nierle 1988).

**Fig. 1:**
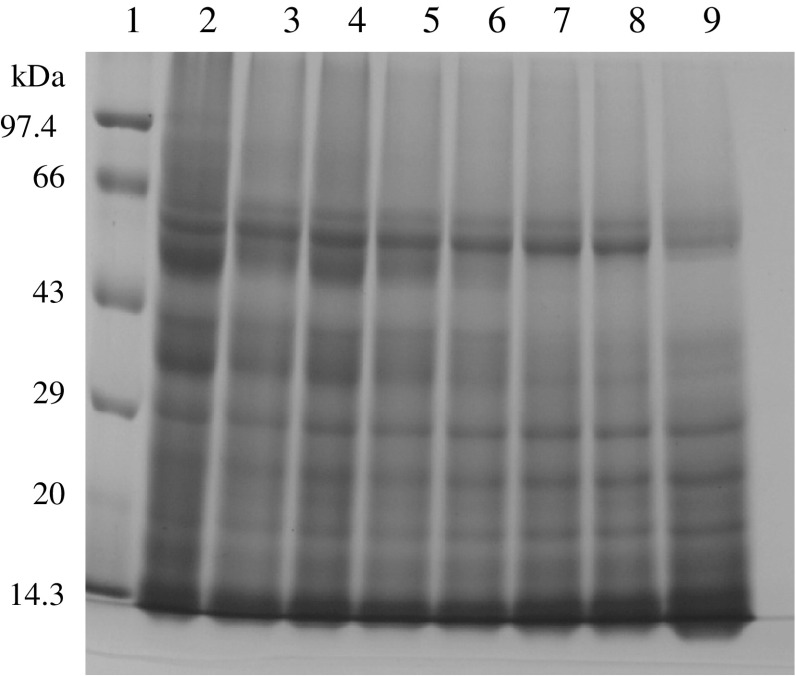
SDS–PAGE of wheat proteins (gliadins) of dough. *Lane 1*: Molecular weight markers (phosphorylase b 97.4 kDa, bovine serum albumin 66 kDa, ovalbumin 43 kDa, carbonic anhydrase 29 kDa, soybean trypsin inhibitor 20 kDa, lysozyme 14.3 kDa). *Lane 2*: Flour sample. *Lane 3–8*: Fermentation samples taken at different time intervals (2–10 h). *Lane 9*: *Malera* sample

**Fig. 2:**
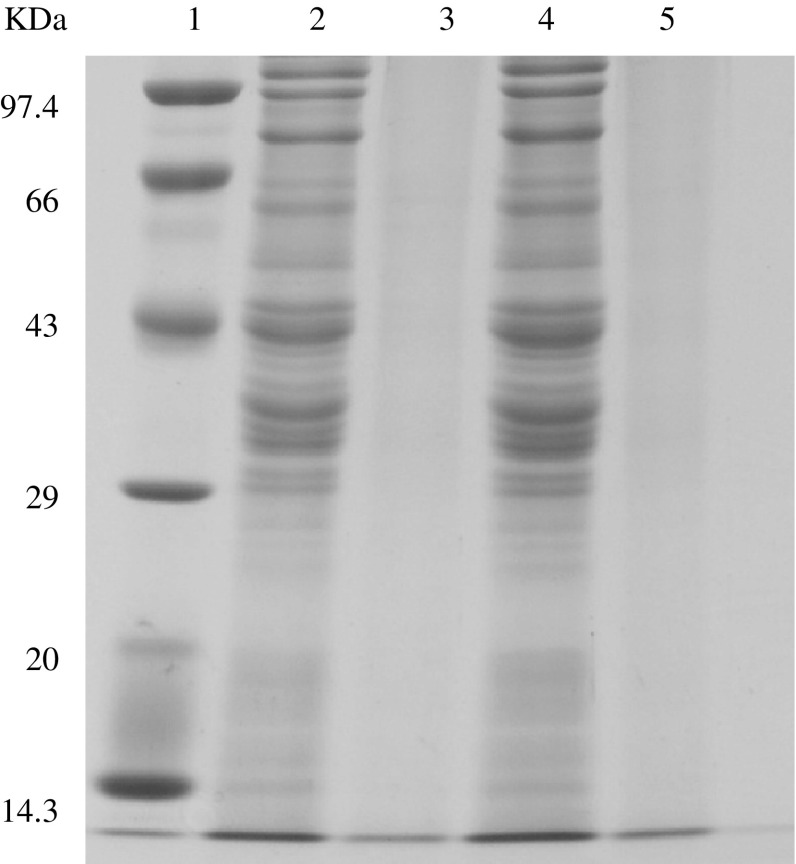
SDS–PAGE of wheat proteins (glutenins) of dough. *Lane 1*: Molecular weight markers (phosphorylase b 97.4 kDa, bovine serum albumin 66 kDa, ovalbumin 43 kDa, carbonic anhydrase 29 kDa, soybean trypsin inhibitor 20 kDa, lysozyme 14.3 kDa). *Lane 2* and *4*: Glutenin samples extracted from different flour samples. *Lane 3* and *5*: Glutenin samples extracted from 10 h of fermented dough during *bhatooru* fermentation

It has also been observed that the electrophoretic profiles of the fermented dough are different from that of unfermented or flour samples. During *bhatooru* fermentation, wheat proteins (gliadins and glutenins) had got hydrolysed due to proteolytic activity of flour and microorganisms (Fig. 1, 2). The disappearance of protein (especially glutenins subunits) bands in the fermented dough were observed, which may be due to the hydrolysis of both HMW (high molecular weight) and LMW (low molecular weight) gluten subunits and α, β, γ and ω subunits of gliadins during fermentation by the activity of proteolytic enzymes. The hydrolysis of wheat proteins with the progress of fermentation can be correlated to the increase in amino acid level in the fermented dough. The substantial hydrolysis of gliadin and glutenin proteins occur during sourdough fermentation due to pH-mediated activation of cereal enzymes, especially aspartic proteinase that appears to be active in the conditions of wheat sourdough (Thiele et al. 2003; Loponen et al. 2004). Furthermore, sourdough fermentation results in solubilisation and depolymerization of the gluten macropolymer (Thiele et al. 2004).

The amylolytic activity was measured up to 10 h of fermentation in dough and control samples and the results are given in Table 2. It first increased from 65.0 to 79.4 U/g of dry matter in 6 h and then decreased to 69.9 U/g of dry matter by 10 h of fermentation. Amylase activity was lower i.e. 33.1 units/g in case of control. Increase in amylolytic enzymes during the course of fermentation has been reported in several Indian fermented foods such as Punjabi *warri, idli, dosa, jalebi, khaman* (Soni and Arora 2000, 1990; Sankaran 1998).

### Vitamin and amino acid analysis

The fermentation of dough significantly enhanced the B vitamin levels especially thiamine, riboflavin and nicotinic acid (Table 3). The rise in the level of various vitamins especially thiamine and riboflavin appears to be due to the increase the microflora and yeasts, most of which have the ability to produce vitamins from simple precursors (Steinkraus 1998). Soni and Arora (2000) also reported significant increase in the water-soluble B vitamins including thiamine, riboflavin and cyanocobalamin in *bhalle* fermentation and *dosa* batter fermentation. *Warri* fermentation also brings about an appreciable rise in water-soluble B vitamins including thiamine, riboflavin and cyanocobalamin (Soni and Arora 2000).

**Table 3:** Vitamin and amino acid content in wheat flour, *malera* and fermented dough

	Flour	*Malera*	Fermented dough
Vitamins (mg/g)			
Thiamine (per 100 g)	0.52 ± 0.026	2.7 ± 0.26	1.57 ± 0.07
Riboflavin	0.003 ± 0.00017	0.005 ± 0.0003	0.081 ± 0.002
Nicotinic acid	0.051 ± 0.0045	0.97 ± 0.02	0.65 ± 0.02
Cyanocobalamin	0.006 ± 0.0002	0.008 ± 0.0002	0.057 ± 0.003
Amino acid (mg/g)			
Methionine	2.6 ± 0.26	5.80.3 ± 0.1	5.7 ± 0.06
Phenylalanine	2.8 ± 0.2	6.6 ± 0.3	6.0 ± 0.08
Threonine	1.6 ± 0.1	4.6 ± 0.17	4.7 ± 0.07
Lysine	1.2 ± 0.2	3.2 ± 0.26	2.3 ± 0.2
Leucine	4.9 ± 0.17	5.6 ± 0.26	5.5 ± 0.2

Values are mean ± SD of three observations

It was observed that fermentation significantly modified the relative amount of amino acids in dough. The various essential amino acids such as methionine, phenylalanine, threonine, leucine and lysine exhibited remarkable increase (Table 3). However, control did not show an appreciable increase in amino acid content with respect to the wheat flour. The increase in these amino acids in *bhatooru* fermentation is an indication of hydrolysis of proteins by the activities of proteolytic enzymes as well as the addition of such amino acids by the fermentative microbes, due to their metabolic activities in the product. Similar findings were reported by Soni and Arora (2000) during the *warri*, *idli* and *dosa* fermentation where they observed a significant increase in free amino acids from 9.79–45.15 mg, 8.3–12.9 mg and 10.2–17.8 mg respectively on dry weight basis. The increase in free d- and l-amino acids in sourdoughs by various lactic acid bacteria and yeasts has been reported by Gobbetti et al. (1994).

The final products *bhatooru* and *chapati*/*roti* prepared from fermented and unfermented dough were analyzed for various biochemical parameters and results are summarized in Table 4. From these studies it can be concluded that nutritive value (especially vitamins and amino acids) in *bhatooru* which was prepared by fermentation with the addition of traditional inoculum (*malera*), is greatly enhanced as compared to *chapati*/*roti*.

**Table 4:** Comparative analysis of *bhatooru* and *roti* (*chapati*)

Parameters	*Bhatooru*	*Roti*
pH	6.00 ± 0.03	7.06 ± 0.06
Total acidity (%)	0.024 ± 0.003	–
Proteins (%)	6.2 ± 0.02	5.1 ± 0.17
Total sugars (%)	62.8 ± 0.83	66.2 ± 1.2
Reducing sugars (mg/g)	11.0 ± 0.36	13.0 ± 1.2
Starch (%)	47.2 ± 1.3	65.0 ± 1.7
Amylase (U/g)	0.180 ± 0.005	ND
Protease (U/g)	ND	ND
Thiamine (mg/100 g)	1.3 ± 0.03	0.49 ± 0.02
Riboflavin (mg/g)	0.041 ± 0.002	0.001 ± 0.0001
Nicotinic acid (mg/g)	0.021 ± 0.002	0.011 ± 0.0017
Cyanocobalamin (mg/g)	0.029 ± 0.003	0.002 ± 0.0002
Methionine (mg/g)	1.4 ± 0.03	1.1 ± 0.036
Threonine (mg/g)	1.4 ± 0.02	1.0 ± 0.07
Phenylalanine (mg/g)	2.1 ± 0.3	1.5 ± 0.026
Lysine (mg/g)	0.72 ± 0.04	0.56 ± 0.036

Values are mean ± SD of three observations

## Conclusions

From these studies it is evident that traditional fermentation adds quality to staple food *bhatooru* by way of enhancing their protein content, vitamin and essential amino acids. This is the first ever study on *bhatooru*, a traditional fermented food of rural hilly people of Himachal which shows that it has better nutritional value as compared to *chapatti/roti* prepared from unfermented dough. So the use of this fermented food as a source of protein, vitamins and amino acids can be popularized to improve the nutrition and social well-being of rural and urban people of Himachal Pradesh. In addition to this, these studies will provide base for scale up of processes practiced at household level to small-scale industrial units by the entrepreneurs.
